# “One of the Weakest Budget Players in the State”: State Funding of Higher Education at the Onset of the COVID-19 Pandemic

**DOI:** 10.3102/01623737231168812

**Published:** 2023-05-08

**Authors:** Denisa Gándara, Meredith S. Billings, Paul G. Rubin, Lindsey Hammond

**Affiliations:** The University of Texas at Austin; Sam Houston State University; The University of Utah; North Carolina State University

**Keywords:** policy, politics, postsecondary education, finance, case studies, qualitative research, state policy, state budgets

## Abstract

Prior studies have documented the pattern of decreased state funding for higher education in periods of economic contraction (i.e., the balance wheel phenomenon). This qualitative case study examines how policymakers in California and Texas made decisions about funding higher education at the onset of the COVID-19 pandemic, when policymakers faced an economic downturn. Data comprise 28 interviews with key state actors and 69 documents. The analysis expands prior understandings of how state policymakers make budgeting decisions that affect higher education by exploring how they perceive certain target populations as deserving or undeserving of state support. The study also sheds light on the tenuous relationship between policymakers’ views of higher education and their funding decisions.

During economic downturns, state funding for public higher education contracts (State Higher Education Executive Officers [SHEEO], 2021). Consistent with these trends, higher education is often viewed as the balance wheel for state budgets, receiving deeper cuts than other areas of state budgets during economic downturns and renewed investment when the economy improves (Delaney & Doyle, 2011, 2018; Hovey, 1999). Reductions in state support for higher education have compounded over time as funding for higher education often does not return to pre-recession levels before the next recession starts (Laderman, 2015). For example, in 2019, average educational appropriations per full-time equivalent student were 8.7% lower than the funding level high before the Great Recession (Weeden, 2019).

In 2020, states forecasted budget shortfalls of $75 billion and $125 billion relative to their pre-COVID projections in 2021 and 2022, respectively (Dadayan, 2020), leading most states to cut higher education funding further. One year after the start of the COVID-19 pandemic, appropriations for higher education had declined in 35 states (Laderman & Tandberg, 2021). In response, Congress passed a series of federal stimulus bills starting in March 2020 with the Coronavirus Aid, Relief, and Economic Security (CARES) Act and ending in March 2021 with the American Rescue Plan that allocated money to higher education (U.S. Department of Education, n.d.-a, 2020). In the first stimulus bill, higher education received approximately $14 billion through the Higher Education Emergency Relief Fund (HEERF; U.S. Department of Education, 2020). In addition, state governors received roughly $3 billion through the Governor’s Emergency Education Relief Fund (GEER Fund) that they could spend on higher education (U.S. Department of Education, n.d.-b). HEERF awarded dollars to institutions and included spending requirements and maintenance-of-effort provisions. In contrast, GEER Funds were more flexible and allocated at the discretion of state governors.

Amid this backdrop, this study addresses the following research question: What explains policymakers’ funding decisions for higher education when facing the COVID-19 economic downturn? We are especially interested in understanding policymakers’ decisions about (a) funding higher education versus other state budget categories (e.g., K–12) and (b) funding higher education institutions (in the form of appropriations) versus students (in the form of financial aid). Framed by a theory of social construction and policy design (Schneider & Ingram, 1993, 1997) and the balance-wheel model (Delaney & Doyle, 2011; Hovey, 1999), we examined higher education funding decisions at the onset of the COVID-19 pandemic in two states that have large and diverse populations but vary in their dominant political ideologies: California and Texas. We used a multiple case study design (Yin, 2017) and drew on data from 28 interviews with key state policy actors and 69 relevant documents.

This study expands our understanding of state funding for higher education in several ways. First, we explored state funding decisions made in the context of a public health crisis. The anticipated COVID-19 downturn and the Great Recession that preceded it were both marked by high levels of budgetary uncertainty and provided access to federal stimulus funds that could offer economic relief to higher education. Despite these similarities, the funding decisions made during the pandemic likely differ from those made in previous economic downturns because of the unique characteristics and cause of the more recent event. During the COVID-19 pandemic, states mandated lockdowns to contain the spread of severe acute respiratory syndrome coronavirus 2 (SARS-CoV-2), negatively affecting state economies (Bauer et al., 2020; Kerns, 2020). The pandemic also had a distinct effect on higher education institutions’ enrollments and finances, which led to unique fiscal needs for these institutions (Kelchen et al., 2021).

Second, this study is one of the few qualitative perspectives that sheds light on the balance-wheel phenomenon. Previous research on higher education funding is overwhelmingly quantitative (e.g., Chatterji et al., 2018; Foster & Fowles, 2018; Li, 2017; McLendon et al., 2009; Taylor et al., 2020). By employing a qualitative multiple case study design, we glean the perspectives of key actors involved in higher education funding decisions while accounting for critical contextual factors surrounding each case.

Third, this study explores policymakers’ attitudes toward higher education and the degree to which their stated views coincide with their funding decisions. Recent public opinion polls suggest that Americans have increasingly negative views on higher education institutions, especially 4-year universities (Fishman et al., 2019), particularly among Republicans (Fingerhut, 2017). However, the growing public support for “free college” (Campaign for Free College Tuition, 2020), which benefits students, suggests that the public may view college *students* as deserving of public benefits (Bell, 2020). Trends from prior recessions suggest policymakers may be responsive to public opinion, as state-sponsored student aid has fared better than higher education appropriations during economic downturns (Li & Zumeta, 2019). However, we know little about policymakers’ views on higher education and the degree to which those views align with their funding decisions. We extend prior research by exploring how policymakers perceive potential beneficiaries of state funding for higher education (i.e., different groups of higher education institutions and students) as either deserving or undeserving of support, and how those views correspond to their state higher education funding decisions.

## Literature Review

This study builds upon research on state policymakers’ higher education funding decisions. In our study and this literature review, we focus on two major types of state funding for higher education, appropriations to higher education institutions and student financial aid. These bodies of literature offer insights that guide our analytic framework on state policymakers’ funding decisions in anticipation of an economic downturn.

Researchers have long been interested in understanding the determinants of state-level higher education funding decisions (Hearn & Ness, 2018). Prior studies have identified various state-level factors associated with appropriations for higher education, including political party control (Archibald & Feldman, 2006; Li, 2017; McLendon et al., 2009; Weerts & Ronca, 2006, 2012), state economic characteristics (Li, 2017; Weerts & Ronca, 2006), legislative professionalism (Lowry, 2016; McLendon et al., 2009), gubernatorial power (McLendon et al., 2009), social ties between legislators and their states’ universities (Chatterji et al., 2018), state demographics (Foster & Fowles, 2018), and higher education governance structures (Lowry, 2016; Tandberg, 2013; Tandberg et al., 2017). Moving beyond internal state factors, researchers have also found that *interstate* factors, such as cross-state policy emulation and advocacy by intermediary organizations, may also influence policymakers’ decisions on funding higher education (Cohen-Vogel et al., 2008; Gándara et al., 2017; P. G. Rubin & Hearn, 2018).

A handful of studies have also examined factors associated with variation in state spending on student financial aid (Bell et al., 2020; Doyle, 2012; Foster & Fowles, 2018). These studies suggest that state-level financial aid funding decisions are largely driven by different factors than appropriations, with notable exceptions. In one study, Doyle (2012) examined predictors of funding for appropriations and state-sponsored financial aid separately. Findings from that study indicate that spending on financial aid was positively related to states’ levels of legislative professionalism (not tested for in the model predicting appropriations) and student enrollments in the private higher education sector (not significant in the model predicting appropriations). On the other hand, government ideology did not predict levels of state financial aid funding, although it was a significant predictor of appropriations (Doyle, 2012).

Despite these differences, prior research hints at one potential factor driving funding for both appropriations and financial aid: the racial composition of the student population (Foster & Fowles, 2018; Hamilton & Nielsen, 2021; Taylor et al., 2020). Foster and Fowles (2018) found that decreases in the state’s White population are associated with reduced spending on appropriations and student financial aid. This finding resonates with Taylor et al. (2020), who found that when White students are overrepresented in a state’s institutions, unified Republican governments spend more on higher education than Democratic or divided governments. These studies suggest policymakers’ higher education funding decisions may be driven by perceptions of deservingness that differ across student demographics and political parties.

In summary, the prior literature, which is almost exclusively quantitative, has identified numerous factors that predict state funding for higher education. Some factors predict funding for state appropriations but not financial aid (e.g., political variables) and vice versa (e.g., enrollment in the private sector); one factor that appears to be associated with both types of funding is the racial composition of the student body. Building on this research, the present study seeks to illuminate why policymakers made certain decisions related to state funding for higher education institutions and college students in anticipation of an economic downturn.

## Conceptual Framework

This study is anchored in the theory of social construction and policy design (Schneider & Ingram, 1993, 1997) and draws insights from the balance-wheel model. Schneider and Ingram’s (1993, 2019) theory scaffolds our understanding of how policymakers distribute benefits and burdens to groups based on policymakers’ perceptions of (a) the groups’ *social constructions*, or how the public views members of the groups as either deserving or undeserving of benefits or burdens and (b) the groups’ relative *political power* (e.g., money, mobilization potential, and access to elected officials). The balance-wheel model, which complements the theory to comprise our conceptual framework, posits that higher education will be disproportionately burdened through state budgeting decisions during economic downturns relative to other areas of the state budget (Delaney & Doyle, 2011, 2018; Hovey, 1999). Through this lens, we examined whether and why policymakers decided to cut funding for higher education disproportionately relative to other state budget categories and why policymakers directed benefits to some target groups within higher education (e.g., students) over others (e.g., higher education institutions).

### Theory of Social Construction and Policy Design

The theory of social construction and policy design posits that policymakers are chiefly driven by their interest in reelection and will be especially responsive to the views of individuals who have influence over their reelection (Schneider & Ingram, 2019). Accordingly, policymakers will seek to benefit groups with more positive social constructions and higher levels of political power (e.g., veterans) and burden those with more negative social constructions and lower levels of political power (e.g., welfare cheats; Schneider & Ingram, 1993). Schneider and Ingram (1993) categorize target populations, or groups targeted by a policy, into four types: advantaged, contenders, dependents, and deviants. *Advantaged* groups have higher levels of political power and more positive social constructions (e.g., medical doctors). The theory predicts that these groups will primarily receive benefits from policy. *Contenders* also have high levels of political power but more negative social constructions (e.g., for-profit colleges). According to the theory, these groups will receive benefits, but policymakers will seek to hide these benefits from the public.

In contrast to contenders, *dependents* tend to have lower levels of political power and more positive social constructions (e.g., individuals with disabilities). The theory anticipates that these groups will receive symbolic benefits but few substantive or material benefits. Finally, *deviants* have negative social constructions and low levels of political power (e.g., terrorists). The theory predicts that deviants will receive burdens through policy. In our analysis, we also consider racialized variation within each of these groups; for instance, while Kreitzer and Smith (2018) categorize low-income families as dependents, other research shows that racially minoritized low-income families are viewed more negatively—and treated less favorably—than White low-income families (Soss et al., 2011).

In our study, target groups within higher education that could receive benefits or burdens from policymakers include various groups of *students* (e.g., low-income, high-achieving, and racially minoritized students) and different types of *higher education institutions* (e.g., community colleges, flagships, and regional comprehensive universities). We chose to focus on these target groups as they are the primary beneficiaries of the two major types of state funding for higher education, student financial aid and appropriations.

#### Students as Target Populations

Research has shown that college students have a positive social construction; the public generally views them as deserving of benefits (Kreitzer & Smith, 2018; Schneider & Ingram, 2019). Yet, based on previous research (e.g., Bell, 2020, 2021; Schneider & Ingram, 2019), we expect perceptions of deservingness to vary across students with different characteristics (e.g., students with high academic achievement versus those with financial need; Bell, 2020). Studies have also shown that members of the public view racially minoritized individuals as less deserving of benefits (Bell, 2021; Schneider & Ingram, 2019).

With respect to political power, previous studies have shown that students can influence policy when they serve in positions of formal power (Rall & Galan, 2022) and when they organize (Bovill et al., 2021; Morgan & Davis, 2019; Rhoads, 2016). For example, the movement that led to President Biden’s executive action on student loan forgiveness suggests that students effectively exert political pressure on policymakers (Krietzberg & Nova, 2022). In addition, research has shown that groups that advocate on behalf of students can influence higher education funding decisions (Gándara, 2016).

#### Higher Education Institutions as Target Populations

Few studies have examined the social construction and political power of colleges and universities. In a study that crowdsourced perceptions of deservingness for numerous politically relevant populations, Kreitzer and Smith (2018) found that for-profit colleges are viewed as having low deservingness but high levels of political power. Similarly, a qualitative study to understand how two states designed performance-based funding policies for higher education found that flagship institutions had relatively high levels of formal political power and a relatively negative social construction (Gándara, 2016).

Prior research also suggests that higher education institutions differ in their levels of political power. A study that examined the process of designing a new state higher education funding model in Colorado found that rural institutions had lower levels of political power, whereas flagships and open-access institutions had higher levels of power (Gándara, 2020). The flagships drew their political power primarily from ties between institutional leaders and elected officials (e.g., former policymakers served as university governmental relations officials) and from their financial resources. Open-access institutions derived their political power from the Speaker of the House’s support for these institutions and from student and community organizing around the issue of equitable state funding for higher education (Gándara, 2020). A quantitative study also sheds light on factors that contribute to different levels of political power across higher education institutions, finding that institutions with higher shares of legislators who are alumni get more state funding (Chatterji et al., 2018). The study’s authors found that this relationship was stronger when the legislator’s alma mater was geographically close to the legislator’s district, suggesting that legislators will advocate for funding for the institutions they attended and those in or near their legislative districts.

### Balance-Wheel Model

The United States has experienced a series of recessions in the last several decades, including in 1990–1991, 2001, 2007–2009, and a seemingly short-lived recession ushered in by the COVID-19 pandemic in 2020 (Bauer et al., 2020). Following each of these recessions, state revenues declined, leading policymakers to decide which areas of the state budget to cut and by how much. The balance-wheel model posits that fiscal deficits will trigger lower spending on higher education (Hovey, 1999). Specifically, this model predicts that higher education will be cut more than other state budget areas in bad budget years and will enjoy greater investment than other budget areas in good budget years (Delaney & Doyle, 2011). Empirical tests of this model support the balance-wheel hypothesis (Delaney & Doyle, 2011, 2018).

Researchers studying the balance-wheel hypothesis have proposed possible explanations for these patterns, including K–12 and Medicaid funding requirements and the ability of higher education to secure nonstate funds (Delaney & Doyle, 2018). Li and Zumeta (2019) used the theory of social construction and policy design and quantitative methods to examine whether and under what circumstances funding for student aid increased at a higher rate than appropriations during economic downturns. Our analysis sheds further light on their findings, which showed that states prioritize funding for student aid during economic downturns. Specifically, our qualitative study empirically analyzes the authors’ premise that higher levels of student aid spending are related to positive social constructions of students. Guided by our conceptual framework, we examine how target groups are socially constructed and how these social constructions interact with their political power to influence higher education funding decisions.

## Research Design

This study employs a multiple case study design (Yin, 2017), drawing on two cases to garner insights into state-level higher education funding decisions. A case-study design allows for an in-depth examination of a phenomenon and is especially apt for answering “how” and “why” questions (Yin, 2017). Using this design, we examined the process surrounding higher education funding decisions at the start of the COVID-19 pandemic in California and Texas.

Case selection followed a theoretical replication logic (Yin, 2017). We selected states expected to differ in policymakers’ funding decisions based on the majority political ideology represented in the state. Case selection was informed by Schneider and Ingram’s (2019) proposition that policymakers will make decisions based on the anticipated responses of their constituents. Perceptions of the values and goals of higher education are increasingly partisan (Doyle, 2007; Fishman et al., 2019). Policymakers’ perceptions of their constituents’ views on higher education, which track with political party affiliation, could influence their funding decisions. We also selected states with similar state-level financial aid portfolios (a mix of need- and merit-based aid) to examine how political partisanship could relate to policymakers’ perceptions of potential beneficiaries of financial aid and their decisions to fund state financial aid programs.

Based on our selection criteria, California and Texas emerged as cases of interest. These states had contrasting political ideologies (California was predominantly liberal, whereas Texas was largely conservative) and similar state-level financial aid portfolios. They also represented the two largest postsecondary enrollments nationally and served a racially and ethnically diverse student population, which allowed us to explore perceptions of deservingness from state policymakers across racial/ethnic student groups. These two states also experienced divergent trends in FY 2021 (Laderman & Tandberg, 2021), with California introducing the largest cut to higher education nationally and Texas minimizing funding cuts by using federal stimulus funds. We explore these differences in funding levels in our description of the cases. Other state characteristics that were relevant for this analysis are presented in Supplemental Table S1 in the online version of the journal.

### Data

Data for this study included 28 interviews with individuals familiar with state higher education funding decisions. Participants included elected officials, legislative staff members, higher education agency officials, higher education institution- and system-level leaders, and staff from state intermediary organizations. For our analyses, we relied primarily on interviews with elected officials, their staff members, and higher education agency officials since we expected them to have the greatest insights on the reasons why policymakers chose to benefit some groups and burden others through their higher education funding decisions. Interviews with other participants primarily served to construct the case summaries and contextualize the findings. Supplemental Table S2 in the online version of the journal includes the number and type of participants by state.

The interviews ranged in length between 30 and 60 minutes and occurred between August 2020 and February 2021. This period allowed us to capture perspectives on policymakers’ decision-making in the context of initial budget shocks, preceding changes that occurred after the budget impacted by the pandemic was adopted. The interviews followed a semi-structured protocol with relevant probes (H. J. Rubin & Rubin, 2011), including questions about revisions made to the FY 2020 to 2021 budgets as well as proposed changes to the subsequent budget in each state.^1^ Guided by our conceptual framework, we also asked about potential explanations for the balance-wheel phenomenon, including why higher education tends to receive steeper cuts than other state budget categories during economic downturns; reasons for differences in funding support for various types of higher education institutions and groups of college students; and perceptions of deservingness of different target populations within higher education.

Our study also drew insights from 69 documents, which included state budgets, news articles, and state executive orders, from June 2020 to February 2021. The documents informed our interview protocols by shedding light on states’ higher education funding decisions before and through the pandemic. In addition, we used the documents to supplement the interview data to construct the case summaries and contextualize the findings.

### Analysis Methods

To analyze the data, we first employed established methods of coding qualitative data (Miles et al., 2018). We used Dedoose, a qualitative and mixed-methods data analysis program, to systematically code interview transcripts. All four authors analyzed the data; two authors focused primarily on California and the other two focused on Texas, although all four authors analyzed transcripts in both states to familiarize ourselves with both cases.

The analysis started with an a priori coding structure. Initial codes were developed based on an analytical framework that included concepts from our literature review, conceptual framework, and guiding research questions (e.g., *state appropriations*, *statewide financial aid*, *uniqueness of COVID-19*, *views on higher education*, *target populations*, *social constructions*, *political power*, and *state-level characteristics*). We then expanded the analytic framework with emergent codes, such as *competition and collaboration across institutions* and *federal funding* (Saldaña, 2016). The full list of parent and child codes is listed in Supplemental Appendix in the online version of the journal.

To enhance the trustworthiness of the results, multiple members of the research team coded the same transcript in the early stages of analysis to develop shared interpretations of the codes and augment the consistency in our coding across interviews and researchers (Saldaña, 2016; Yin, 2017). Throughout the coding process, we held peer debriefing sessions to discuss how we were coding, recommend new codes, organize (or reorganize) the coding structure, and triangulate our data sources (using multiple interviews and documents). During peer debriefs, we also discussed memos, which we created in Dedoose to note theoretically informed findings (e.g., social construction of different types of institutions), emergent findings (e.g., higher education has alternative sources of funding), unexpected findings (e.g., generally positive views on higher education), and converging and contradictory evidence across data sources.

Consistent with our multiple case-study design (Yin, 2017), we first conducted within-case analyses. Two researchers developed case summaries and identified key findings for their respective states. Case summaries are presented in the next section. The key findings from California were the following: institutions have alternative sources of revenue; there is no constitutional obligation to fund higher education; college affordability is imperative, especially for historically underrepresented students. In Texas, the key findings include: the discretionary nature of funding higher education; a desire to maintain college access and persistence amid the pandemic; the value of higher education for the workforce, especially given the economic contraction; and policymakers distributing funding to institutions in their own districts.

After identifying key findings for our respective states, we revisited the data with the following aims: to construct the themes, to find evidence supporting or challenging the themes, and to identify similarities and differences between the two states. Specifically, the two researchers who led the initial coding process in each state reanalyzed the data in their respective states, focusing on the codes related to the findings identified in the within-case analyses. Throughout the analysis process, we considered disconfirming evidence and alternative explanations. For example, we considered that funding decisions may be driven by factors other than target groups’ social constructions or political power (e.g., constitutional constraints). Before presenting the thematic findings, we summarize the two cases, focusing on the relevant state context and decisions policymakers made related to funding for higher education at the onset of the COVID-19 pandemic.

## Case Summaries

### California Case

California has three public higher education systems: the University of California (UC) System with 10 universities, the California State University (CSU) System with 23 universities, and the California Community Colleges (CCC) System with 116 colleges. The sector is guided by the California Master Plan for Higher Education of 1960, which designates a division of labor among the three systems and offers a framework for each system to pursue access with excellence. Despite its initial utility, the Master Plan has been criticized in recent years as outdated, as it has struggled to adapt to the state’s changing needs, leading to policy misalignments and a fragmented approach to postsecondary education (Odle & Finney, 2020).

With respect to state funding for higher education, the legislature determines levels of appropriations for the university systems but not for community colleges. The appropriations process for community colleges is guided by Proposition 98, which amended the state’s constitution to guarantee a minimum level of funding to K–12 schools and community colleges. Specifically, community colleges and the K–12 system are guaranteed at least 40% of the general fund under Proposition 98, with about 11% allocated to community colleges (Public Policy Institute of California, n.d.). If community colleges do not receive their full share due to deferrals, the state is required to pay the remaining portion in subsequent fiscal years.

Higher education institutions in California were poised to see increased funding before the pandemic. The FY 2021 state budget included a 5% increase for the UC and CSU systems (California Department of Finance, 2020b). Following the onset of the COVID-19 pandemic and subsequent revenue declines in the state, the May budget revision reduced funding for UC and CSU by 15%, including a withdrawal of the 5% increase and a new 10% cut to partially compensate for the state budget shortfall (Burke & Gordon, 2020; California Department of Finance, 2020c, 2020d). In the final version of the budget, UC and CSU experienced a budget cut of 12% to 13% (California Department of Finance, 2020a).

For the CCC system, the state passed a budget that allowed the system to spend the same level as in FY 2020, but the system was given $1.45 billion in deferrals because the state could not meet its funding commitment for the year (Fensterwald, 2020; Stavely et al., 2020). State officials presented funding cuts and deferrals as subject to reversals if the state received sufficient federal funds by October 2020 (California Department of Finance, 2020c). As expected, the federal funding did not materialize, solidifying the cuts and deferrals.

California also funds higher education through its financial aid programs. The largest program in the state is the Cal Grant, which has entitlement awards for high school seniors, recent high school graduates, and community college transfer students. These programs guarantee funding to students who meet the eligibility requirements, which include demonstrated financial need and a minimum grade point average (GPA) in high school or community college, depending on the award (California Student Aid Commission [CSAC], n.d.-b). If students are ineligible for the entitlement awards, they can apply for the competitive Cal Grant program, which awards aid according to students’ financial need and GPA (CSAC, n.d.-a). The Cal Grant program also has awards for current and former foster care youth and for students pursuing occupational or technical programs (CSAC, n.d.-b).

California policymakers prioritized preserving funding for the state’s financial aid programs. However, Governor Newsom did not allocate any of the GEER Funds included in the CARES Act to higher education. Instead, those funds were used entirely for K–12, primarily to offset expenses in the transition to online learning. As a state finance official explained, there were concerns about “[K12] learning loss . . . [particularly] with a massive shift to remote learning.” As illustrated in this quote and described in more detail in Rubin et al. (2022), K–12 funding was prioritized over postsecondary education in the allocation of GEER Funds because policymakers believed K–12 needed more financial resources to support students with virtual schooling compared with higher education.

### Texas Case

Texas’s public higher education ecosystem is more complex than California’s; it includes 37 4-year universities, 3 state colleges, 50 community (junior) college districts, 6 technical colleges, and 10 health-related institutions. Each of the five sectors of the state has a separate funding formula. The Texas Higher Education Coordinating Board (THECB) makes recommendations for distributing state funds to individual institutions or systems. However, state legislators determine levels of appropriations for higher education.

Texas has three statewide financial aid programs, all primarily of need-based. The largest aid program is the Toward Excellence, Access, and Success (TEXAS) Grants. Since 2013, the TEXAS Grants program has had a merit component; state aid is first awarded to students with financial need who have met certain academic criteria. The other two state aid programs are the Texas Educational Opportunity Grant (TEOG), a need-based aid program for students attending public, 2-year colleges, technical colleges, or state colleges, and the Tuition Equalization Grant, which can be used at private, nonprofit colleges and universities in Texas.

The pandemic disrupted a period of economic prosperity in Texas. In 2019, the state projected an 8.1% increase in state revenue for general spending in FY 2020 to 2021. Consistent with the balance-wheel hypothesis and similar to California, before the pandemic, state legislators had indicated their intention to boost investment in higher education during the following legislative session. Unfortunately, the state’s economy took a double hit in 2020, with a steep decline in oil and gas revenues coupled with the pandemic-induced economic contraction, which led Texas to face a revenue shortfall of $4.58 billion (Korte, 2020).

In May 2020, the governor, lieutenant governor, and Speaker of the House sent a letter to state agencies, including higher education institutions and the THECB, asking them to reduce their budgets for FY 2020 to 2021 by 5%. This request only applied to general academic institutions; 2-year colleges and health-related institutions were exempt. The state maintained this request for FY 2022 to 2023, but it was set to apply to all institutions except community (junior) colleges.^2^

Unlike in California, the Texas governor chose to allocate some GEER Funds ($175 million) to higher education (Office of the Texas Governor, 2020). These funds were used for five primary purposes: (a) to offset funding cuts to the statewide financial aid programs; (b) to provide emergency financial aid (to be disbursed by institutions); (c) to create a program to reskill/upskill displaced workers; (d) to enhance the availability of open educational resources; and (e) to update and expand the student data infrastructure. At the conclusion of our analysis in March 2021, both state chambers had recommended maintaining higher education appropriations for the 2022 to 2023 biennium at the 2020 to 2021 appropriated levels.

In summary, the onset of the COVID-19 pandemic catalyzed funding cuts for higher education in both California and Texas, albeit to varying degrees across the states and across sectors. This study illuminates the patterns that emerged in higher education funding decisions (e.g., community colleges fared better than 4-year universities; students fared better than institutions). The findings reveal the extent to which patterns track with expectations from the balance-wheel model and the theory of social construction and policy design and potential explanations for deviations from these expectations.

## Thematic Findings

We present four cross-state themes from our analysis of policymakers’ higher education funding decisions. The first two themes capture two primary reasons why higher education institutions tend to be cut disproportionately during economic downturns: the *inability of higher education to compete with other state budget categories* and *higher education institutions’ access to other sources of revenue*. The third theme describes policymakers’ *preferences for funding students* (through financial aid) over institutions (through appropriations). These preferences are rooted in perceptions that students deserve benefits and that they (and perhaps their parents) have political power manifested as advocacy, constituent calls, and voting potential. The fourth theme describes the *complex relationship between policymakers’ views on higher education and their funding decisions*.

### “Higher Ed Is Not . . . as Sexy”: Higher Education Versus Other State Budget Categories

In both states, policymakers mentioned that higher education struggles to compete with other state budget categories given legal obligations and federal matching incentives tied to other areas of the budget. In California, funding for UC and CSU is not constitutionally protected, whereas funding levels for community colleges are determined pursuant to Proposition 98. Community colleges in California are thus insulated from major funding disruptions. This protection was attributed in part to the coalition of community colleges with K–12, which gives community colleges a source of political power. Most participants felt that UC and CSU are a lower budgetary priority than health and human services (HHS), K–12 education, and community colleges. Since a substantial segment of the California state budget is subject to federal or state constitutional restrictions, an elected official explained that “a large portion of the budget is on autopilot, so there’s not much for higher ed to compete with.” According to this elected official, higher education’s lack of guaranteed funding makes them “one of the weakest budget players in the state,” even though they are “universally beloved.” This quote, which is representative of sentiments we heard in both states, captures a perception that the lower priority given to higher education (which refers to UC and CSU in California) relative to other budget categories does not reflect negative will toward this sector.

In Texas, numerous participants also mentioned the state’s legal obligations to funding K–12, including in response to court challenges. One legislator contrasted the state’s obligations to K–12 with policymakers’ discretion over higher education:

[In K12], it’s been codified in general law that you have to spend certain amounts of money . . . That’s not the case for higher ed. So in higher ed, there’s formulas and there’s traditions and there’s previous practices . . . but everything is up for grabs.

Echoing comments from legislators in both states, a higher education official in Texas observed that legislators have greater discretion over funding for higher education relative to other budget items: “once you exclude [K12 education] and you exclude things that they have less control over like Medicaid and debt service and employee benefits, there’s not . . . a lot of general revenue in your general state government agencies.”

Beyond legal obligations, some participants mentioned that funding HHS takes priority, in part because of federal matching incentives. A former legislator in Texas who was a higher education leader at the time of data collection explained,

kind of a rule of thumb, when you’re looking at places to pull back spending, [is] you try not to sacrifice the things for which there’s a federal match . . . because you don’t want to pull $1 out to give up $2 of federal matching money . . .

Policymakers and other participants underscored these practical considerations as key constraints on funding decisions.

Policymakers’ decisions about funding higher education also seemed to be influenced by their perceptions of the social constructions of different target populations (i.e., how they thought the public viewed certain groups as more or less deserving of benefits or burdens). The analysis captured the social construction of groups within higher education and other areas of the state budget, including K–12. In one illustrative quote, a legislator in Texas described how the confluence of practical and political factors results in higher education getting “squeezed” between K–12 and HHS:

Obviously, [K12] gets a lot more attention. We’re talking about children, we’re talking about property taxes, we’re talking about parents, their kids, their money. I mean, it’s explosive, right . . . So, as a general rule, the effect of that is higher ed . . . tends to be squeezed between [HHS and K12] . . . So you’ve got one driven by . . . disease and illness . . . And the other’s driven by general law, minimum requirements of spending, political pressure, great political and media pressure if you don’t [fund it]. So higher ed tends to get squeezed between those two . . . What happens to higher ed is not as, as interesting to the media, and it’s not as emotional. It’s not as sexy.

This quote illustrates how both social constructions and the political power of target populations play a role in policymakers’ decisions to privilege K–12 and HHS in state budget decisions. Children and those who are ill tend to be viewed as deserving of policy benefits (Kreitzer & Smith, 2018). Beyond their positive social constructions, K–12 beneficiaries enjoy political power through their ability to exert “political pressure,” including through “the media.” Illustrating the intersection between social constructions and target populations, this quote suggests the media is interested in covering issues related to K–12 funding (a manifestation of political power) *because* K–12 target populations are widely viewed as deserving, making it an issue that is “emotional.”

Most of the participants referenced K–12 and HHS as the primary sectors competing with higher education for state funds. However, a Texas university system official and former legislator also mentioned corrections. They noted that

sometimes . . . when you’re looking at higher ed [in the budget] . . . it falls down a few rungs on the ladder . . . so when you’re talking about giving Department of Public Safety officers raises, you know, that’s not an entitlement, it’s not something you *have* to do. But, you know, by god, you’re probably going to do that.

They concluded that policymakers’ perceptions that certain groups should receive more benefits limit the share of the budget available for other discretionary items, such as higher education: “. . . when you do look at the levels of discretionary cash that as a lawmaker you actually have some input in, that is pretty limited.” This quote suggests that policymakers are constrained in their budgeting decisions by both structural limitations (e.g., legal obligations) and expectations that they will fund certain target populations that are viewed as highly deserving or as having a high degree of political power (e.g., police officers in Texas).

In summary, policymakers make decisions about funding higher education based on the perceptions of the deservingness of higher education beneficiaries (both institutions and students) relative to other budget categories and their stakeholders (e.g., “children,” “parents,” people who are sick). Findings also suggest that K–12 target populations may have higher levels of political power, including the power of media attention. Although not mentioned explicitly, K–12 and HHS also have broader constituencies than higher education, which could afford them greater political power in the form of a larger voting bloc and more widespread advocacy. State policymakers are also constrained by legal obligations and, to some extent, the availability of federal matching funds, which restrict the funding available for higher education. These findings suggest that the distribution of benefits and burdens through policy is limited by other factors, including legal restrictions and federalist incentives. Findings also suggest that funding decisions that are unfavorable to higher education do not necessarily reflect a disdain for this sector. We elaborate on this finding in the final theme.

### “Most Institutions Have a Little Bit of Leeway”: Reserves and Access to Other Revenues

The second explanation policymakers offered for the balance-wheel phenomenon is that higher education institutions have access to other sources of revenue, unlike other areas in the state budget. In alignment with our theory and prior work on the factors associated with perceptions of deservingness (Kreitzer et al., 2022; Oorschot, 2000), policymakers viewed higher education institutions as less deserving of state support than other sectors because they had lower levels of need, given their access to nonstate funding streams. In Texas, a legislator explained, “there is an assumption . . . but even a reality that unlike other places in the [state] budget, universities do have an alternative source of revenue, whether it’s through athletics or . . . tuition, or research grant dollars.” Corroborating this perspective, a higher education official in Texas noted, “And to be fair for legislators . . . if they cut us, we can increase tuition.”

Beyond tuition revenues, participants in both states mentioned other sources of income available to higher education, including philanthropy and revenue from auxiliaries, and local tax revenue for community colleges. Unique to the COVID-19 pandemic, higher education institutions also had access to federal stimulus funding through the CARES Act. In both states, a few respondents described a strategy to combine federal and state funding to manage the budget cuts. A Texas state agency official explained that they used either GEER Funds or state dollars to backfill the cuts to the TEXAS Grants, depending on students’ eligibility for federal financial aid. A Texas community college leader explained how federal stimulus funds allowed them to ask the state for help funding workforce development programs since the CARES Act would partially cover other pressing needs, such as student aid.

The narrative about higher education institutions’ access to other sources of revenue also captured policymakers’ perceptions that some colleges and universities were well-resourced, at least relative to other state budget categories. A staff member on one of the legislative budget committees in California noted that CSU and UC had “big reserves” and “it was kinda hard to imagine like protecting these giant institutions that had endowments and reserves while we were cutting courts and the court services.” Similarly, a legislative staff member explained,

if you are going to cut higher education, it’s a little bit easier and more nebulous when you cut an institution . . . they have reserves, they have some ability to shift some funds, you know there’s the sense that typically most institutions have a little bit of leeway.

Specific to COVID-related reductions, a finance official in California explained,

We were aware [that] both UC and CSU had reserve funding available to cover [a] contingency such as this. So . . . I think we were well-aware that they were modestly positioned to adopt and . . . implement the reductions that were coming through.

This finding highlights the association between policymakers’ perceptions of higher education institutions’ levels of need and their deservingness of state support.

Findings related to policymakers’ perceptions of higher education institutions’ levels of need also illuminate differences between different types of institutions. For instance, although UC and CSU were discussed together in some statements, as in the previous quotes, policymakers also distinguished between the systems in their funding decisions.

Illustratively, UC and CSU had been subject to the same dollar increases or decreases until the enactment of a recent change subjecting the systems to the same *percent* changes in the budget. This change benefits CSU, as they receive more total state appropriations than UC. Several participants confirmed that the change was, in fact, intended to benefit CSU. When asked why the shift was made, a legislative staffer remarked that “UC tended to be in trouble with the legislature more often over the last 10 years,” and “there were a few years where the legislature just decided that we like CSU better.” The staffer rationalized that this change made sense because CSU has “more students” and

UC has this gigantic budget that includes medical centers and national labs, and they just have so much more revenue. Now most of that is kind of restricted but I think in general, [UCs] do have more flexibility. They obviously have a lot more philanthropy, for example, than CSU. So, I think it makes sense that we’ve made that switch.

This example demonstrates how policymakers’ preference for CSU (“we like CSU better”)—a form of political power—coupled with their view that CSU is more deserving and in need of support than UC played a role in the distribution of benefits to CSU.

Perceptions of CSU’s deservingness were also tied to perceptions of the students they serve. One legislative staff member described why they tend to allocate more financial aid to CSU, namely “because we see them as a broader access institution that serves more students of color.” A legislative staffer also explained that it “is harder for us as the legislature to understand what we’re buying when we give UC money versus with CSU,” as the legislature is more interested in “how many more students can we cram into the campus to get in versus like okay let’s fund this kind of nebulous research project.” This quote illustrates how policymakers’ views that CSU deserved support were partly fueled by their preference for funding instruction (over research and other activities).

Similarly to CSU, community colleges were largely viewed as having greater need and thus deserving of more state support. In response to a question about why community colleges were exempt from the budget reduction in Texas, a legislator replied, “probably because . . . community colleges are already struggling to stay afloat . . . and the governor, legislators have heard that very loudly.” This quote suggests community colleges lobbied effectively, a sign of political power. Corroborating this finding, community college officials discussed the benefits of engaging directly with coordinating board officials, legislators, and governor’s office staff members. One community college leader stated, “we spent a lot of time talking [to] our local legislators.” They proceeded to mention that one of those legislators holds a powerful post on a committee with jurisdiction over higher education funding decisions.

Beyond perceptions that community colleges are underfunded, reasons for supporting community colleges in both states included the role they play in workforce development and their low prices. Some policymakers also described community colleges as “lean,” especially in contrast to some 4-year universities, which contributed to their support for the sector.

In California, community colleges were primarily spared due to constitutional protections (Rubin et al., 2022). However, three participants (a legislative staff member, an intermediary official, and an academic) mentioned another possible factor shaping policymakers’ support for these institutions in California: their perception that community colleges can play a role in promoting racial equity. Similar to comments about CSU, a staff member from a nonpartisan education organization observed that community colleges’ “political clout is growing . . . probably much more because of . . . racial justice and . . . equity concerns in the state.” This comment suggests that policymakers’ support for community colleges in California may be increasing. At the same time, an expert on education policy in California posited that the power of UC and CSU may be decreasing, claiming that they are “not as powerful as [they] used to be.” This participant observed that “in the old days,” legislators supported their alma maters. However, “that’s long faded out and the minority groups that have grown in power in California, they’re angry with the [UC and CSU] systems because they don’t think they get admitted . . .” These quotes suggest that racially minoritized groups may have swelling political power in California that has shaped perceptions of UC (and possibly CSU) as inaccessible. These constructions may have resulted in declining state support for these institutions.

As described previously, community colleges were poised to fare relatively well in budget decisions at the onset of the pandemic. In California, community college funding is protected through Proposition 98. In Texas, community colleges were exempted from the 5% budget reduction applied to state agencies. A legislative staff member in Texas also predicted that 2-year colleges would be a priority in the following legislative session.

In summary, policymakers’ decisions about funding higher education institutions are shaped by the practical reality that colleges and universities have access to other sources of revenue. However, they are also influenced by the perception that higher education institutions are generally well-resourced and better positioned to weather a weak economy than other state agencies. Despite these views, participants generally recognized differences in resources and access to funding between institution types. Perceptions that CSUs and community colleges are underfunded and thus deserving of state support translated to some funding decisions that favored them. Our findings also show how policymakers’ perceptions of CSUs in California and community colleges in both states are shaped by effective advocacy on behalf of these institutions. These institutions’ political power (access to policymakers and effective lobbying) enables them to influence policymakers’ perceptions of the degree to which they are deserving of funding. In California, we also heard that racial equity concerns may play a role in higher education funding decisions. Community colleges (and perhaps CSUs) may benefit from widespread perceptions that these institutions can advance racial justice goals and from political power tied to racial justice movements in the state.

### Supporting Students “Is Like Apple Pie”: Preferences for Funding Financial Aid

In both states, policymakers chose to protect funding for financial aid in state budgets. The preferences of policymakers for funding financial aid appeared especially pronounced in the context of the pandemic, given the drops in postsecondary enrollments and persistence rates and the perceived need to upskill and reskill state residents. Most of the expected losses to higher education funding came in the form of state appropriations to institutions, a trend that resembles those observed in previous recessions. A California government staff member predicted, “My suspicion is we will continue to cut the institutions and not the students,” adding “that’s both probably the right thing to do, but also the politically right thing to do.” Participants shared two primary reasons for policymakers’ decisions to preserve statewide financial aid. The first was the imperative to improve college access and postsecondary attainment, primarily for economic reasons. The second, which is captured in the previous quote, was political pragmatism anchored in policymakers’ expectation that funding students would yield political (electoral) benefits.

Speaking to policymakers’ interest in *promoting college access and attainment*, a Texas legislator noted, “the single most important thing when it comes to higher ed is to not let a generation of people fall into the cracks that otherwise might be pursuing higher ed.” In another representative quote, a former legislator likewise described broad support for financial aid to enhance higher education access:

I’ll go back to TEXAS Grants and TEOGs as a way to make sure that we help as many people as we can reasonably help to get into higher education . . . Even for your . . . most far right, conservatives, they’ll find themselves in the minority, in terms of pushing back on that . . . So the majority of your . . . Democrats and most of your mainstream Republicans are going to . . . recognize that that’s an important thing for us to continue to support.

This quote captures the perception of this former legislator that support for expanding college access is broad and spans across political parties, with rare exceptions at the right end of the political spectrum. Implicit in these comments is the power of a hidden target population: employers, which previous research has described as advantaged (having positive social constructions and high political power; Newton, 2005). The importance of not letting “a generation of people fall [through] the cracks” appeals to the economic imperative of having a populace with the knowledge and skills to fill workforce (and employers’) needs.

The second major explanation for policymakers’ support for financial aid is the political pragmatism associated with funding students. This finding captures policymakers’ perceptions of college students’ social constructions (as deserving of state support) and their political power (potential votes and political pressure, including constituent calls). Exemplifying the political power of students, a higher education official in California shared,

So far there’s been a commitment to protect [financial aid]. A lot of advocacy in the state of California to protect affordability, with a group of students, many of whom are financially vulnerable . . . So far . . . [there’s been] a pretty significant political commitment to maintain those programs.

Participants across roles and states converged in their perceptions that funding students was politically popular. In a representative quote, a community college official in Texas observed that “helping students go to college is like apple pie . . . It’s really doing the right thing, everybody feels good about it . . . putting the money out to help students is very politically correct.” Sharing a similar sentiment, a higher education official in California called college affordability “the fourth rail of politics,” and another stated, “politically, the position here in California is that all of our students are deserving [of aid] . . .” These quotes capture general perceptions that college students deserve state support.

Other findings capture the perceptions of policymakers that they would gain political benefits from increasing financial aid (and incur political costs from cutting it). A Texas legislator observed,

As a political matter, you get credit for . . . supporting students. So if you put [funding] into the TEXAS Grants program . . . you can say, well, we increased student financial aid . . . And it’s not only, not only it’s good for students, because they’re hurting, it’s a recession and their parents are hurting. But it’s also good politics, if you will.

A state legislative staff member in California also mentioned how perceptions of deservingness of target groups influence policymakers’ decisions to protect financial aid: “. . . cutting students directly, cutting families directly, working class families. It’s just something that no one wants to do . . .” Policymakers also contrasted the political costs and benefits of funding financial aid versus appropriations. For instance, a Texas legislator described it as

A headline, a political reality . . . because TEXAS Grants, people know what that means. If you cut . . . [the] UT System, no one really knows what that means . . . My district could be like, “Oh, you didn’t fund [the] UT System? What does that mean? . . . [It] doesn’t even affect me.”

By referencing “a headline,” this quote alludes to the role of the media in providing political power to TEXAS Grants beneficiaries. Corroborating this finding, a higher education official observed, “I don’t think higher ed, it doesn’t have a constituency. So what if you vote against higher ed? So what?” A complementary perspective, which we explore in the final theme, is that legislators treat institutions in their legislative districts as constituents.

Two findings on the social construction of students were unique to California. First, positive portrayals of students seemed to be reserved for residents of the state. Multiple participants mentioned public perceptions that out-of-state residents were crowding academically high-achieving Californians out of the UCs. This perspective was captured in a claim that instead of serving Californians, the UCs were admitting “5,000 people from China” and “all these people from Maryland.” Second, as noted previously, California policymakers were especially attuned to the needs of historically underserved students, including those who are racially minoritized. A state legislative staff member in California posited that, “you cannot cut anything where students of color or low-income students, underrepresented students will be disproportionately hurt . . .” A state finance official contextualized this equity imperative, given the disproportionate impacts of COVID-19 on “underrepresented” communities:

In the environment that we were seeing in the state [and] just globally . . . we know that some of the most impacted individuals for COVID in the state’s economy are . . . underrepresented students, are underrepresented families, are low-income families, so we made . . . it very clear that we would be protecting that program from any reductions.

As described in the previous section, some participants postulated that in California, racially minoritized people have increasing levels of political power, as suggested by California’s ban on the ACT and SAT on the grounds that they are racially and ethnically discriminatory. Complicating this picture, however, a California education policy expert noted that “Whites and Asians are still [the] majority . . . of the electorate in terms of [being] registered to vote . . .” Illustrating this complex and shifting political landscape in California, following this interview, voters elected to keep a ban on affirmative action in the state. That decision underscores the limits of political power among racially minoritized groups in California.

In summary, policymakers described two primary reasons for preserving financial aid: expanding college access and attainment and the political pragmatism of supporting students. The former reason captures a practical factor that influences policymakers’ funding decisions. It also sheds light on a hidden target population, employers, who benefit from increasing college access and attainment. The latter reason (political pragmatism) coincides with the theory of social construction and policy design, capturing policymakers’ views that the public views college students as deserving of state support and expectations that their decisions regarding financial aid have political consequences. Political consequences may result from unrest among students themselves or their parents, who policymakers may view as constituting an important voting bloc. This theme also captures some differentiation in perceptions of deservingness between student groups, primarily in California.

### “Practical Politics”: Policymakers’ Views of Higher Education and Funding Decisions

The final theme captures the complex relationship between policymakers’ views on higher education and their funding decisions. General goodwill toward higher education did not seem to drive funding decisions affecting this sector. Relationships between higher education officials and policymakers emerged as a more salient factor in these decisions.

Policymakers in our study generally expressed positive views on higher education overall, especially given its role in economic development, but also offered some critiques, mostly directed at 4-year universities. An underlying message in our findings is that policymakers primarily value the instructional mission of higher education, with less support for non-instructional spending (including “admin and research”). Positive perceptions of community colleges and CSUs were tied to the role these institutions play in educating students.

Our findings suggest that policymakers’ broad views on higher education rarely influence their funding decisions. Other factors mentioned in previous themes, such as the discretionary nature of higher education funding and “pragmatic” issues related to the workforce, appeared more prominently in discussions about reasons for higher education funding decisions than policymakers’ overarching views about higher education. In addition, decisions to fund specific institutions were largely shaped by personal ties between policymakers and those institutions.

A staff member at a nonpartisan educational organization in California described the importance of these relationships, which are a form of political power, in the ability of higher education “to be successful in the budgeting process.” They continued, “Those relationships and the ability to work those relationships pay dividends for higher ed . . . I think folks underestimate how far that takes negotiations.” Illustrating this point, they speculated that the change in leadership at UC would hamper the system’s ability to advocate successfully for state funding.

As previously described, CSUs were privileged in recent funding decisions, even though they were subject to COVID-related budget cuts. In Texas, community colleges were exempted from the 5% across-the-board reduction. These decisions were attributed in part to the support of individual policymakers for community colleges and CSUs. In Texas, a participant with significant knowledge of the funding process credited the Speaker of the House, who was highly supportive of community colleges, with the decision to exempt these institutions from cuts. Likewise, in California, some participants suggested that Governor Newsom was especially supportive of the CSUs; as lieutenant governor, he had been active on the CSU board. One CSU leader described the value of this relationship with the governor:

He understands us, he’s been in a lot of the conversations . . . He gets it. And . . . he knows all the work that we’ve been trying to do on Graduation Initiative 2025 . . . He knows our unique value . . . in social mobility and . . . what we’re doing for communities. And I think those are all aligned to his own values, both personally and professionally, politically. So . . . I think it’s going to be really helpful . . . There’s going to be cuts . . . and I think it’s going to be really difficult for him to [cut us].

As this quote illustrates, policymakers’ perceptions of institutions and their willingness to support them are partly shaped by their relationships with representatives from those institutions. Higher education officials described how they leveraged personal relationships with elected officials and their staff members to increase state support for their institutions. These relationships translate to political power by yielding direct access to policymakers who can advocate on their behalf and vote in favor of benefits for those institutions.

Participants also shared their perceptions that elected officials make decisions based on personal connections to institutions, including as alumni or as parents of children attending those institutions. For example, a nonpartisan education organizational staff member in California observed,

there are lots of folks who went to [UC] in the legislature, so they have strong personal ties to the system, especially UC Berkeley. So, I think that drives a lot of how folks feel about funding to the segments . . . you have a bunch of alum in the [legislature] . . . I think that sort of drives the narrative about what should be funded and what’s valuable.

In Texas, a community college official shared a similar view when describing their perceptions of how responsive policymakers are to requests from different segments of higher education:

Then the politics come in. And your elected officials are not alumni from the community colleges. They are alumni from the major university . . . graduates of UT and A&M . . . [are] very prevalent in our legislature. So when they need funding, I think the legislature is almost much more responsive, whereas a community college less so. Very much less so . . . community colleges have always been at the bottom of the pecking order.

On one hand, community colleges in Texas and CSUs in California seem to have the most positive social constructions in their respective states. On the other hand, they do not benefit from the same type of political power as the flagships. This would suggest that these widely appreciated institutions can be at a disadvantage. However, findings from this study suggest that at least at the onset of the COVID-19 pandemic, community colleges in Texas and CSUs in California had advocates in powerful positions, such as the Speaker of the House in Texas and the governor in California, which translated to funding decisions that were favorable to these institutions.

The analysis also uncovered another reason why policymakers choose to support individual higher education institutions: their desire to extend benefits to local constituencies in their legislative districts. This finding was almost exclusively evident in Texas. In an illustrative quote, a legislator remarked,

higher education, more than just about anything other than I guess I’d say [K12], [is] very much driven by what’s going on in your own district and to the exclusion of even thinking of higher education as a whole . . .

A legislative staff member corroborated this view, describing state funding for higher education in Texas as “parochial.” Similarly, a university official in Texas posited that policymakers’ preference for benefiting their district “will sometimes trump maybe their . . . philosophical view of the value of higher education . . . It’s a very, very practical politics versus kind of like their esoteric idea of the value of higher ed.”

To illustrate the “parochial” nature of higher education funding in Texas, a legislator told a story of legislators seizing an opportunity to fund higher education in their districts, declaring that “everybody wanted to bring home some bacon,” resulting in a “feeding frenzy.” In another example, a legislator described another “very conservative” legislator who questioned the value of funding higher education generally but “really pushed” for funding for an institution in their district. This legislator concluded, “So I do think there is a lot of that provincial attitude that’s impacting this.” This comment also highlights the tenuous relationship between policymakers’ personal views on higher education and their funding decisions. These findings align with Schneider and Ingram’s (2019) contention that policymakers’ decisions will be motivated by their expectations of how their constituencies will respond to the policy changes. They anticipate that electoral rewards will follow their extension of benefits to their constituents.

A related finding, which appeared in both states, is that higher education institutions or systems benefit from having physical campuses across the state since this gives them representation from multiple legislators. In California, a state legislative staffer observed, “CSU’s advantage is definitely that they’re just better represented throughout the state . . . so their reach is broader.” Similarly, in Texas a higher education official shared, “you know, almost every member has a community college in their district. So . . . there’s a broad base for community colleges, a lot of times, because, you know, everyone has one. They’re everywhere.” In contrast, a higher education official shared their perception that institutions in less populated areas are disadvantaged by having fewer legislators representing the districts in which they are located.

In summary, findings suggest that policymakers’ higher education funding decisions were driven largely by their interest in benefiting institutions with which they have a relationship (e.g., as former alumni) and in benefiting their own districts rather than overarching views on the degree to which higher education is deserving of state funding. Flagships benefited from political power manifested as close ties to legislators. Community colleges and CSUs, for their part, benefited from having representatives across that state and from having elected officials who supported them in key political positions, including the governorship, the House Speakership, and key positions on powerful legislative committees.

## Discussion and Policy Implications

Absent the ability or will to raise revenues, economic downturns force state policymakers to decide which areas of the state budget to cut and by how much. When this happens, higher education institutions are typically targeted for steeper cuts (Delaney & Doyle, 2011; Doyle & Delaney, 2009; Hovey, 1999). This study contextualizes the trends we observe related to state funding for higher education during economic downturns by examining why policymakers made certain decisions at the onset of the COVID-19 pandemic.

The theory of social construction and policy design guided our analysis of the reasons policymakers made decisions that benefited some groups within higher education and burdened others. Our findings both corroborate and extend prior understandings of state higher education funding decisions, especially during an economic downturn. As others have observed, higher education funding is limited by spending requirements for other budget categories (Delaney & Doyle, 2018); policymakers also recognize that higher education institutions have access to other sources of revenue. Extending previous work on state higher education funding decisions during economic downturns, this study shows how perceptions of different groups’ deservingness and political power shaped policymakers’ decisions. Although policymakers described higher education as deserving of state support, they portrayed beneficiaries of K–12 and HHS as *more* deserving of state funding than higher education stakeholders. These findings suggest that the distribution of benefits in budgeting decisions may depend on the social constructions of certain target groups *relative to* the construction of other groups.

Political power, the other dimension of the theoretical framework, did not emerge as an important factor shaping policymakers’ decisions to fund higher education over other segments of the state budget, although it was relevant for differences between higher education institutions and groups of students. It is possible that the larger constituencies of K–12 and HHS compared with higher education contribute to greater political power for these segments, although participants did not mention this in interviews. The findings also suggest that the media may give K–12 higher levels of political power. By bringing attention to K–12 funding issues, the media may play a role in exerting political pressure to make decisions that are beneficial to K–12. These findings add to previous work on the role of the media (informing the public or constructing political spectacle) in educational policy and politics (Anderson, 2007). Although Schneider and Ingram’s (1993) theory recognizes the role of the media in shaping social constructions of target groups, to our knowledge, our study is the first to shed light on the possibility that the media can affect target groups’ levels of political power by amplifying an issue.

### Different Target Groups Within Higher Education

Our analysis also sheds light on differences in the social construction and political power of various target groups within higher education and how the two dimensions of the theory relate to policymakers’ funding decisions. Beginning with *community colleges*, funding for these institutions was protected in both states. In California, funding was preserved due to Proposition 98, a constitutional amendment that established a minimum level of funding for K–12 schools and community colleges. As some participants noted, through Proposition 98, community colleges benefited from their coalition with K–12, a form of political power. Although community colleges in California were generally viewed favorably (as accessible and serving students historically underserved in higher education), these positive social constructions played a lesser role than constitutional protections in higher education funding decisions in California.

In Texas, community colleges were exempted from the 5% reduction. That decision was tied to policymakers’ perceptions that community colleges had high levels of need and played an instrumental role in meeting the state’s economic needs. Policymakers’ views on community colleges were shaped by community college leaders, who had ties to legislators. Although flagships also had high levels of political power in Texas, they enjoyed less positive social constructions than community colleges. Community college leaders’ personal ties to policymakers, coupled with policymakers’ perceptions that the public views these institutions favorably (and their own positive views of community colleges), helped these institutions avert budget cuts.

Turning to California, funding decisions at the onset of the pandemic were not favorable for *CSU*, as they endured budget cuts. However, a change in allocations that preceded the pandemic did benefit CSU, at the expense of UC. This change was attributed to perceptions that UC “has this gigantic budget” and policymakers’ decision that they “like[d] CSU better.” CSUs were generally portrayed positively, given their broad access, focus on student success, production of bachelor’s degrees, and perceived role in advancing racial justice. Participants also noted two sources of political power for CSUs, access to the governor and widespread legislative support, given the presence of CSU campuses across the state.

Although not described explicitly, the decision to benefit CSU could also have been influenced by political power among the CSUs and their constituents. In California, policymakers associated CSUs (and community colleges) with another target population, racially minoritized groups. Several participants in California, across various roles, mentioned the burgeoning political power of these groups, fueled by their collective action and the alignment between their racial justice demands and the values of an increasing number of elected officials in the state. While not discussed explicitly, this political power could offer opportunities for advocates to shape policymakers’ perceptions of CSUs and community colleges as deserving of state support.

Our findings related to support for racially minoritized students in California contribute to an emerging literature on the racialized nature of social constructions and political power in educational policy (e.g., Gándara, 2020; Jabbar et al., 2022). Our study took place at the height of the “racial reckoning” that followed the murders, perpetrated by police, of Breonna Taylor, George Floyd, and other Black people (Horowitz et al., 2020). This specific racial context may have amplified the political power of racially minoritized people as well as public perceptions that they are deserving of state support, at least in the more liberal-leaning California.

Notwithstanding positive constructions of community colleges and CSUs, these institutions have historically received lower levels of funding per student than more selective 4-year universities. One possibility is that the benefits extended to these institutions in recent years signify a change in state support for higher education. In other words, policymakers may be increasingly inclined to support less selective and lower resourced public higher education institutions, since they view them as having greater need and playing a more direct role in filling workforce needs. In California, participants mentioned this possibility, suggesting that the political power of UCs may be declining as their social construction worsens.^3^

Alternatively, we may see that more selective institutions will continue to be privileged in state policymakers’ higher education funding decisions, given their high levels of political power. While we found that community colleges and CSUs had political power, some of that power stems from the support of single individuals (e.g., a governor or Speaker of the House), suggesting this power may be less stable than the political power of the flagships, which is derived from multiple ties to elected officials (e.g., alumni). This phenomenon would be consistent with Schneider and Ingram’s (1997) theory, which predicts that dependents (groups with positive social constructions but low political power) will receive symbolic, not material, benefits. Future research building on these case studies can take a longitudinal approach, tracking political power, social constructions, and funding distributions over time and evaluating the relationship between them.

This study also sheds light on policymakers’ support for funding students via financial aid, which is largely based on “political pragmatism.” This finding aligns with an extension of the theory that focuses on anticipatory feedback (Schneider & Ingram, 2019). Our study captures policymakers’ anticipation that the public will reward their support for financial aid. Our findings suggest these expectations are shaped by both social constructions and the political power of financial aid beneficiaries. Policymakers shared their impressions that the public views college students, or those who aspire to higher education, as deserving of state benefits. Moreover, participants shared various sources of political power in the budget process among financial aid beneficiaries, including constituent calls from students and parents and in California, “a lot of advocacy” to keep college affordable.

Our findings related to policymakers’ decisions to fund students directly also shed light on a hidden target population: employers. Policymakers supported student financial aid because that would increase college access, helping the state fill workforce needs. Although not named explicitly, key beneficiaries of these decisions were employers. In line with these findings, previous research has shown how employers and the business community more broadly have advocated for policies that support higher education access and attainment (e.g., Gándara et al., 2017; Gándara & Hearn, 2019).

### The Role of COVID

Some of the findings represent long-standing influences on state higher education funding; others appear more pronounced due to COVID-19. One phenomenon that seems to be unaltered by the pandemic is the finding that higher education struggles to compete with other state budget categories. Moreover, policymakers’ perceptions that higher education institutions have access to other sources of revenue appear unchanged by COVID-19. This point is noteworthy since the pandemic did affect institutions’ ability to generate nonstate revenue due in part to declining enrollments, pressure to disburse tuition and fee refunds, and reduced income from auxiliaries. Another finding that transcends the pandemic is the concern among some California residents and policymakers that the UCs are serving too many non-Californians.

In contrast, the emphasis on funding students seemed especially pronounced due to the pandemic, given higher levels of student need due to weak economic conditions as well as the importance of having an educated population for the states’ economic recoveries. In California, participants also mentioned the importance of supporting students who had been hardest hit by the pandemic, including racially minoritized communities. In this state, some participants speculated that racially minoritized groups had growing levels of political power. However, historical accounts suggest these apparent surges in power among racially minoritized groups are often short-lived and accompanied by White racial backlash (Anderson, 2016).

### Patterns Across States

The two states we selected as cases for this study, California and Texas, are similar in many respects (e.g., population size, demographics, and type of statewide financial aid) but differ in their political ideology and partisanship. As expected, we found some differences between California and Texas that could be related to the two states’ distinct political ideologies. Policymakers in the more liberal-leaning California were more likely to discuss racial equity as a motivation for higher education funding decisions than those in the more conservative Texas. Another difference we observed is that Texas allocated a portion of federal GEER Funds to higher education, whereas California opted to distribute all those funds to K–12. We are unable to determine from our analysis whether these decisions relate to political ideology. Participants credited that decision to governors’ perceptions of relative need across K–12 and higher education, with Governor Newsom deciding that K–12 was in greater need of federal funds than higher education because of the rapid shift to online learning and the accompanying technological and infrastructure costs to support virtual schooling. Our study was not able to explain why California excluded higher education from GEER Funds distributions while Texas did not. California was one of 15 states that did not allocate GEER Funds to higher education (McMorris & Knight, 2022). Future research could examine the reasons for these divergent decisions, including the role of political power and social constructions of K–12 relative to higher education across the states.

We also found similarities across the two politically distinct states. In both Texas and California, policymakers emphasized the economic value of higher education; this was a primary driver of support for the sector. In both states, policymakers expressed broad support for higher education. Another similarity across the states is that they both prioritized preserving financial aid for similar reasons: it is both the “right thing to do” and “politically the right thing to do,” as described by a California state government staffer. Thus, in both states, the political calculations of both sets of policymakers led to similar funding decisions, shielding community colleges from cuts and prioritizing funding for students. These decisions benefited target groups with high levels of political power and positive social constructions.

### Policy Implications

The findings from this analysis yield several policy implications. First, these funding decisions matter since recession-induced cuts to appropriations have led to lower degree completion rates (Deming & Walters, 2018) and higher tuition and fees (Webber, 2017), pricing some students out of higher education or leaving them with debilitating debt burdens (Bleemer et al., 2017). Conversely, higher levels of appropriations for higher education have positive effects on educational attainment (Chakrabarti et al., 2020). Second, the findings illuminate goodwill toward community colleges in both states and CSUs in California. This could signal a policy window to advocate for greater support for these institutions. Third, findings suggest policymakers may uncritically accept higher education institutions’ reliance on nonstate sources of revenue, which could have regressive consequences. Institutions that serve more racially minoritized, first-generation, and low-income students have lesser access to other sources of revenue, including tuition and research dollars. In the context of unregulated tuition in Texas, raising tuition prices can reduce access to higher education for historically underserved students (Flores & Shepherd, 2014), a consideration that is compounded in the current climate, given the disproportionately negative effects of the pandemic on historically underserved students (Gould & Wilson, 2020; National Student Clearinghouse, 2021; Pirtle, 2020).

In conclusion, this study elucidates why certain changes were made in funding higher education at the onset of the COVID-19 pandemic. Future research should examine how decisions differ at later stages of the budget process. Findings from this study can help higher education lobbyists, administrators, advocates, and researchers more fully understand the role that social constructions, political power, and other factors play in higher education funding decisions and how to influence these decisions to lead to more desired outcomes.

## Supplemental Material

sj-pdf-1-epa-10.3102_01623737231168812 – Supplemental material for “One of the Weakest Budget Players in the State”: State Funding of Higher Education at the Onset of the COVID-19 Pandemic Click here for additional data file. Supplemental material, sj-pdf-1-epa-10.3102_01623737231168812 for “One of the Weakest Budget Players in the State”: State Funding of Higher Education at the Onset of the COVID-19 Pandemic by Denisa Gándara, Meredith S. Billings, Paul G. Rubin and Lindsey Hammond in Educational Evaluation and Policy Analysis

## Notes

1. Texas maintains a biennial budget cycle, whereas California’s budget is produced annually. This differing state feature was not significant in our analysis since our focus centered on specific higher education-related budgetary decisions impacted by the onset of the COVID-19 pandemic. The analysis period (August 2020–February 2021) covered a portion of one legislative session in each state.

2. Formula funding for junior colleges was exempt, but non-formula items for these institutions were subject to the reduction.

3. In our study, the UCs were treated as a monolith (both in our questions and in participants’ responses), but recent work illuminates important variations in levels of support for universities within the system (Hamilton & Nielsen, 2021). Future research should attend to these differences within systems.
