# Supplementary material for: “One of the Weakest Budget Players in the State”: State Funding of Higher Education at the Onset of the COVID-19 Pandemic
Source: Educ Eval Policy Anal. 2023 May 8:01623737231168812. doi: 10.3102/01623737231168812 (PMC10170260; doi:10.3102/01623737231168812)
Supplement: sj-pdf-1-epa-10.3102_01623737231168812 – Supplemental material for “One of the Weakest Budget Players in the State”: State Funding of Higher Education at the Onset of the COVID-19 Pandemic [file sj-pdf-1-epa-10.3102_01623737231168812.pdf]

Online Supplemental Appendices

Online Appendix A

Table 1: Characteristics of Case-Study States

	**California**	**Texas**
State Median Age[1]	36.7	34.8
State %Population, 18 Years Old+[1]	77.2%	74.2%
State %Population, Non-White[1]	63.5%	58.8%
Political Party Control	Unified Democrat	Unified Republican
Type of State Financial Aid Offered	Mix: Need and Merit	Mix: Need and Merit
Notable State Financial Aid Programs	Cal Grant<br>Middle Class Scholarship	TEXAS Grant<br>Texas Educational Opportunity Grant Program (TEOG)<br>Tuition Equalization Grant Program (TEG)
Governing Agency	N/A	Texas Higher Education Coordinating Board
Public Higher Education Landscape	10 research-focused campuses (University of California System); 23 four-year public universities (California State University System); 116 community colleges (California Community Colleges System)	6 University Systems (37 four-year institutions); 3 state colleges, 50 community (junior) college districts; 1 technical college system (6 institutions); 10 health-related institutions
2020 State Appropriations per FTE	$9,531	$8,147
2020 Total FTE Enrollment	1,607,937	1,071,308
Budget Cycle	Annual	Biennial

[1] State demographic data come from the 2020 American Community Survey, 5-Year Survey (U.S. Census Bureau, 2022). All other data come from the *State Higher Education Finance: FY2019* report from the State Higher Education Executive Officers Association (SHEEO, 2021).

Table 2: Interview Participants by Position and State

	California	Texas
Elected State Officials	1	4
Non-Elected State Government Staff	5	2
Higher Education Official (State Agency/System/Institution)	4	8
Non-Partisan State Organization Staff	3	-
Other	1	-
Total	14	14

Online Appendix B: Parent and Child Codes

Actors
- Governor
- Higher education agency
- Intermediaries
- Legislators
    - Speaker of the House
- Lieutenant governor
- University / system officials
    - Governmental relations

Collaboration / non-competition among institutions to increase funding
Competition across institutions for funding
Federal government
Incrementalism
Inequities across institutions / within systems
Other funding (beyond state appropriations and financial aid)
- Capital funding
- Career and technical education
- Local tax revenues
- Research funding
- Special items / earmarks
- Tuition / Institutions Raising Tuition
- Hospitals

Political power
- Alumni
- Children of legislators (attending specific institution)
- Institutional geography and representation (in district)
- Personal relationships

Prestige / Rankings
Regional universities tend to be forgotten
Relationship between views on higher education and funding decisions
Social constructions
State appropriations
- Balance wheel
    - Counterevidence (Higher Did OK)
    - Federal matching funds (Healthcare)
    - HEIs have another source of revenue (tuition)
    - Lower priority (relative to other state budget items)
    - No constitutional obligation to fund higher education

State characteristics
- Politics / ideology
- Demographic changes
- Economy
- Governance (e.g., higher education agency)

- Prior decisions / state policies
- State budget process / timing
- Unique to the state
    - California
    - Texas

Statewide financial aid

- Promise programs
- Reasons for funding financial aid
    - Access for underrepresented students
    - College affordability
    - Funding students directly
    - Political pragmatism

Target populations

- Institutions
    - Four-year universities
    - Two-year colleges
- Students
    - Historically underrepresented students / "diverse" students
    - Merit based aid recipients

The Great Recession

- Lessons learned

The uniqueness of COVID-19

- Enrollment trends
- GEER funds
- Higher expenses for institutions (online learning, technology, PPE)
- Lower expenses for institutions / reduced quality
- Reduced revenue at colleges and universities
- Remote working / online meetings
- State budget constraints (COVID-19 specific)

Views on higher education

- “Liberalism gone wild”
- Administrative bloat / inefficient
- Focused on student success
- Healthcare
- Higher ed should be accessible to all
- Higher ed should be more affordable
- Not everyone needs to go to college
- Not serving the state (out of state enrollments)
- Research
    - Some research is useless
    - Valuable contribution
- Workforce / economic development (value of higher education)
